# Circular RNA-protein interactions: functions, mechanisms, and identification

**DOI:** 10.7150/thno.42174

**Published:** 2020-02-10

**Authors:** Anqing Huang, Haoxiao Zheng, Zhiye Wu, Minsheng Chen, Yuli Huang

**Affiliations:** 1 Department of Cardiology, Shunde Hospital, Southern Medical University, Jiazhi Road, Lunjiao Town, Shunde District, Foshan, 528300, China.; 2 Department of Cardiology, Laboratory of Heart Center, Zhujiang Hospital, Southern Medical University, Guangzhou, China.; 3 The George Institute for Global Health, NSW 2042 Australia.

**Keywords:** circular RNAs (circRNAs), RNA binding proteins (RBPs), biogenesis, degradation, translation

## Abstract

Circular RNAs (circRNAs) are covalently closed, endogenous RNAs with no 5′ end caps or 3′ poly(A) tails. These RNAs are expressed in tissue-specific, cell-specific, and developmental stage-specific patterns. The biogenesis of circRNAs is now known to be regulated by multiple specific factors; however, circRNAs were previously thought to be insignificant byproducts of splicing errors. Recent studies have demonstrated their activity as microRNA (miRNA) sponges as well as protein sponges, decoys, scaffolds, and recruiters, and some circRNAs even act as translation templates in multiple pathophysiological processes. CircRNAs bind and sequester specific proteins to appropriate subcellular positions, and they participate in modulating certain protein-protein and protein-RNA interactions. Conversely, several proteins play an indispensable role in the life cycle of circRNAs from biogenesis to degradation. However, the exact mechanisms of these interactions between proteins and circRNAs remain unknown. Here, we review the current knowledge regarding circRNA-protein interactions and the methods used to identify and characterize these interactions. We also summarize new insights into the potential mechanisms underlying these interactions.

## Introduction

Circular RNAs (circRNAs) are covalently closed, endogenous biomolecules with no 5′ end caps or 3′ poly(A) tails, and these RNAs belong to the category of non-coding RNA (ncRNA) molecules 1 that were first discovered in pathogens in the 1970s 2. The term “circRNA” was first coined in 1976 by Sanger and colleagues when they characterized the structure of viroids, which are infectious, single-stranded, covalently closed RNA molecules, but these molecules were ignored in later studies 3 as circRNAs were thought to be the byproducts of splicing errors and to lack significant biological effect. Recently, accompanied by advances in RNA sequencing technologies and bioinformatic approaches 4, scientists have identified thousands of circRNAs in eukaryotes, including in fungi, protists, plants, worms, fish, insects, and mammals, and they have found that these RNAs have tissue-specific, cell-specific, and developmental stage-specific expression patterns 5 and are conserved across species 6.

Since the landmark discovery in 2013 of ciRS-7/CDR1as (circular RNA sponge for miR-7), which functions as a miR-7 sponge, circRNAs have become a hot topic in RNA research 7, 8. In 2017, the first *in vivo* loss-of-function study of ciRS-7/CDR1as in mice revealed that a neuron-specific circRNA regulates sensorimotor gating and synaptic transmission in the brain, providing a novel insight into the biological functions of circRNAs 9. It is now known that circRNAs have a wide range of biological functions from gene expression regulation to protein coding and mRNA competition; this is described further below 1. As a covalently closed circular molecule, circRNAs are more stable than other RNAs. This stability is crucial and will likely prove an ideal property of circRNAs during their future development as biomarkers 10-13. CircRNAs are also proving to be useful molecules as therapeutic targets for multiple diseases, including diabetes mellitus, neurological disorders, cardiovascular diseases, chronic inflammatory diseases, and cancer 5, 14-16.

Although no general function for circRNAs has been identified as yet, some roles have been described and these are shared by subsets of circRNAs, such as those acting as miRNA and protein sponges. There have been several detailed reviews of circRNAs acting as miRNA sponges 17, 18. The patterns of circRNA-protein interactions are, however, more complex and interesting than those of circRNA-miRNA interactions. CircRNA-binding proteins play critical roles in regulating circRNA synthesis and degradation, and circRNA-protein interactions have been reported to influence protein expression, biogenesis, and pathophysiological processes 19, 20. Indeed, circRNAs can bind, store, sort, and sequester proteins to particular subcellular locations. However, the exact mechanisms of circRNA interactions with proteins and other biomolecules mediating their effects have yet to be fully elucidated.

The goal of this review is to provide an overview of the current understanding of circRNA-protein interactions. Here, we summarize new insights into potential mechanisms underlying the biogenesis, biology, and functions of circRNAs, with a particular focus on circRNA-protein interactions, and we outline the approaches used to investigate these circRNA-protein interactions.

## The Characteristics and Biogenesis of circRNAs

### The characteristics of circRNAs

#### Abundance

Large-scale RNA profiling has indicated that approximately 75% of the human genome can be transcribed into RNA 17. In the human brain, 20% of the genes produce circRNAs, whereas in the heart, only approximately 9% of the expressed genes produce circRNAs 20. Furthermore, the abundance of circRNAs is specific to cell type, as they appear to have higher expression levels in low-proliferating cells such as cardiomyocytes compared to the high-proliferating cells of the liver 21. The increased levels of circRNAs observed in the developing heart, lung, and brain tissues appear to be mainly the result of accumulation. Evidence also suggests that there is age-related accumulation of circRNAs in the heart and neural tissues 5, 22. The age-dependent accumulation of circRNAs in the brain is likely due to the high stability of these molecules. Their resistance to exonucleases enables some of these circRNAs to accumulate to relatively high levels. Most circRNAs are generated from pre-mRNAs that also produce linear forms of RNA, and their expression patterns are consistent with those of their host mRNAs 23. Although circRNAs are abundant, they are generally expressed at low levels compared to mRNAs 24. However, some studies have reported that the expression of a circular RNA does not correlate with the expression of its cognate linear mRNA; in fact, under certain circumstances, circRNAs are expressed at a much higher level than their linear counterparts 25, 26.

#### Stability

Although most circRNAs exist in the cytoplasm, they are remarkably stable and resistant to RNase R and other exonucleases because they lack free ends 27. Therefore, circRNAs have a longer half-life than their linear RNA counterparts. The average half-life of circRNAs in cells exceeds 48 h while mRNAs only last on average for 10 h 28. This feature makes circRNAs ideal for serving as biomarkers of multiple diseases including cancer. However, circRNAs may still be sensitive to many other RNases such as RNase A, RNase T1, and RNase T2, which may comprise a crucial pathway for the degradation of circRNAs 29. Interestingly, some circRNAs are cleaved by protein-mediated microRNAs. For example, it has been reported that miR-671 directs the cleavage of a circular antisense transcript of the Cerebellar Degeneration-Related protein 1 (CDR1) locus in an Ago2 slicer-dependent manner 30. In addition to their circular structure, other factors and mechanisms may contribute to the stability of circRNAs but these are still largely unknown.

#### Specificity

Numerous circRNAs exhibit specificity to certain tissues and developmental periods 5, 31, 32. Notably, circRNAs are evolutionarily conserved across species and are present in most organisms, including archaea, plants, yeast, and most metazoans 33. RNA-sequencing of human adult and fetal tissues (heart, kidney, liver, lung, colon, and stomach) have shown that up to 50% of circRNAs are tissue-specific, and both the number and expression levels of different circRNAs in fetal tissues are higher than those in adult tissues 34. CircRNAs are also specific to subcellular locations. While exonic circRNAs are predominantly found in the cytoplasm, other circRNAs such as circular intronic RNAs (ciRNAs) and exon-intron circRNAs (EIciRNAs) are enriched in the nucleus 35. Currently, circRNAs are believed to be produced in the nucleus but it remains unclear how they are transported into the cytoplasm. Some studies have indicated that circRNAs are transported into the cytoplasm by the ATP-dependent RNA helicase in a size-dependent manner 36. The N6-methyladenosine (m6A) modification may also play a role in circRNA transportation. Chen et al. recently identified an m6A-modified circRNA, circNSUN2, which is upregulated in colorectal carcinoma patients, that may enhance the stability of high-mobility group AT-hook protein 2 mRNA to promote colorectal carcinoma metastasis. Furthermore, the m6A modification increases the export of circNSUN2 to the cytoplasm 37. However, the mechanisms for extracellular transportation are not yet fully understood.

#### Translation

Although most circRNAs are not translated, several recent studies have confirmed that certain circRNAs can be translated into proteins. For example, circ-FBXW7 can be translated into the protein FBXW7-185aa, but the functions of this protein are still largely unknown 38.

### The biogenesis of circRNAs

**Figure 1:**
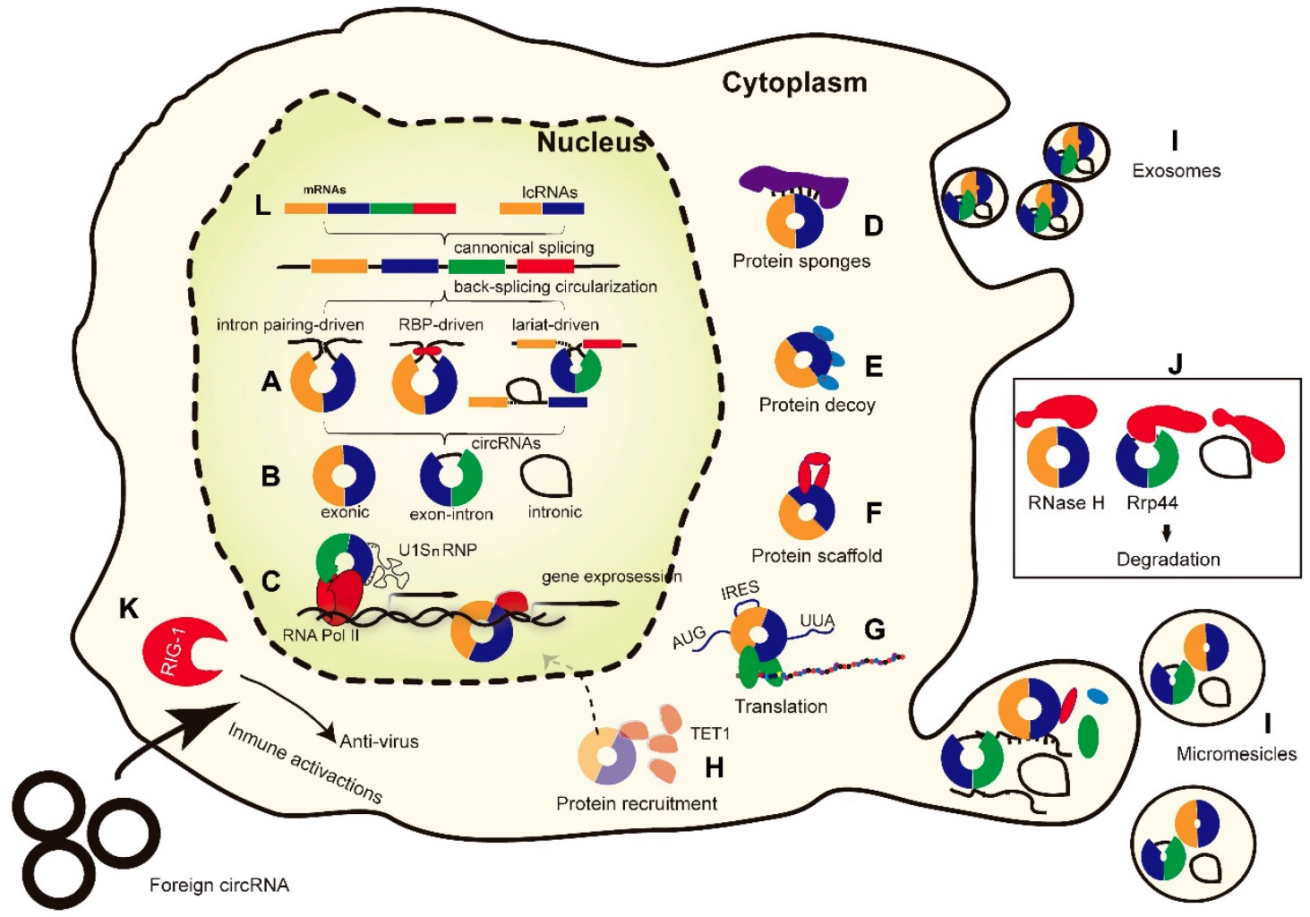
** Proteins play an indispensable role in the life cycle of circRNAs from biogenesis to biological function and degradation. (A-B)** CircRNAs are formed by back-splicing into three major types of circRNA. **(C-H)** The functions of circRNAs in interacting with proteins. Several circRNAs have also been reported to encode proteins. **(I)** CircRNAs may be released from cells into the intracellular environment via exosomes or microvesicles. **(J)** The degradation of circRNAs mediated by RNase. It is still unclear whether the degradation of circRNAs by RNase happened mainly outside or within the cells. **(K)** Exogenous circRNAs can activate the RIG-1 cellular immune response pathway. **(L)** Linear mRNAs and linear non-coding RNAs (lncRNAs) can also be generated from spliceosome-mediated canonical splicing. IRES: internal ribosome entry site. RBPs: RNA-binding proteins. RIG-1: retinoic acid-inducible gene 1 protein. TET1: Tet methylcytosine dioxygenase 1. U1SnRNP: U1 small nuclear ribonucleoprotein.

CircRNAs are generally believed to be derived from canonical splice sites. When pre-mRNA processing events are slowed down, the infant RNAs can be directed to alternative pathways that facilitate back-splicing 23. The main hypothesis of back-splicing is that looping of the intron sequences flanking the downstream splice-donor site and the upstream splice-acceptor site brings these sites into close proximity. The back-spliced junction is a unique characteristic of circRNAs, and it is important in detecting and analyzing the function of circRNAs. Different circRNAs have different junctions, and there are three models of the formation of circRNA loops (**Figure 1A**): **(1)** Intron pairing, where splicing is prompted by reverse complementary sequences such as ALU repeats (located in the upstream and downstream introns), bringing the splice donor site and the upstream splice acceptor site into close proximity to form the loop. Mutational analysis has revealed that ~100 nt of each repeat is sufficient for exon circularization, even when there is competition between canonical splicing and back-splicing 21, 39. Nevertheless, the presence of intronic repeats is not always sufficient to trigger back-splicing. **(2)** Exon skipping and intron lariat formation, where the formation of covalently closed exonic circRNAs is initiated by the 3′ end of an exon (splice donor site) being joined to the 5′ end of the same exon (single-exon circRNA) or to that of an upstream exon (multiple-exon circRNA). **(3)** RNA-binding protein (RBP)-mediated models, where certain *trans*-acting activator RBPs bind specifically to each of the flanking introns, creating a bridge that brings the splice donor and acceptor sites close enough to form a loop 39, 40. The alternative splicing factor Quaking (QKI) binds to flanking introns and forms dimers, thereby bringing the intervening splice sites into close proximity 40. However, the intermediate steps of the circularization are still unknown.

Based on their structural domains and biogenesis features, circRNAs can be classified into three common types (**Figure 1B**): (1) exonic circRNAs (ecircRNAs), which are mainly found in the cytoplasm; (2) circular intronic RNAs (ciRNAs), which are predominantly localized in the nucleus; and (3) exon-intron circRNAs (EIciRNAs), which are mainly found in the nucleus. EcircRNAs appear to be the most abundant circRNA type, accounting for over 80% of known circRNAs 41.

## The Functions of circRNAs

Besides acting as miRNA sponges, several other roles of circRNAs have been proposed (**Figure 1C-I**). By enhancing binding to RNA polymerase II (RNA Pol II), circRNAs located in the nucleus may modulate the transcription of their host genes 42. CircRNAs may also interact with regulatory RBPs, including through their activity as protein sponges, decoys, scaffolds, and recruiters, and further affect the fate of their target mRNAs. Moreover, some circRNAs also contain an internal ribosome entry site and can directly encode proteins.

## CircRNA-protein interactions

While the main function of circRNAs is exerted through their activity as miRNA sponges, their second-most important function is exerted via circRNA-protein interactions.

The most well-known proteins interacting with RNA molecules are the RBPs. RBPs are a class of proteins associated with the metabolic processing of RNAs by mediating their maturation, transport, localization, and translation, and these proteins even participate in forming ribonucleoprotein complexes 43. Many circRNAs are predicted to interact with RBPs through specific binding sites, although bioinformatic analyses of circRNA sequences have predicted very little enrichment in RBP-binding sites. However, recent studies have indicated that RNA-RBP interactions are significantly influenced by the tertiary structure of the RNA molecules. Thus, the unique tertiary structure of circRNAs may exert an effect on their protein-binding capacity separate to that of the traditional nucleotide sequence-based mode of binding, and which mode of binding is used may depend on specific circumstances.

The binding of circRNAs to proteins may have bidirectional effects. RNA-protein interactions have been reported to influence protein expression and function, while also regulating circRNA synthesis and degradation. CircRNAs can serve as protein sponges or decoys to influence their cellular functions, thereby regulating gene transcription, inhibiting cell cycle progression, promoting cardiac senescence, inducing apoptosis, and promoting proliferation and cell survival among other processes (**Table 1**). These functions are described in further detail below.

### CircRNAs regulate protein expression and function

EIciRNAs can reduce the transcription of their parental gene via interaction with host U1 snRNP and RNA Pol II, thereby competing with mRNA production and consequently affecting protein translation 54. Similarly, Abdelmohsen et al. reported competition between a circRNA (circPABPN1) and its cognate mRNA for the RNA-binding protein, HuR, which influenced protein expression 53. Another study showed that circANRIL impairs pre-rRNA processing and ribosomal function by binding to Pescadillo homolog 1 (PES1), an essential 60S pre-ribosomal assembly factor, in human vascular smooth muscle cells and macrophages, resulting in the activation of p53 47. These results indicated that circRNAs also regulate the role of ribosomes in protein expression.

Furthermore, circRNAs can influence the binding of proteins in a positive feedback loop. Ashwal-Fluss et al. showed that circ-Mbl contains several binding sites for the MBL protein and regulates MBL expression in a positive feedback manner from the *MBL* locus itself 54.

### Proteins regulate circRNA synthesis and degradation

#### Synthesis

CircRNAs are transcribed by RNA Pol II and are generated by the spliceosome, which includes heterogeneous nuclear ribonucleoproteins (hnRNPs) such as the small ribonucleoprotein particle U1 subunits 70K and C (snRNP-U1-70K and snRNP-U1-C, respectively), and the SR proteins 60. CircRNA biogenesis is likely to be regulated by RBPs, transcription factors, and a combination of *cis*-acting elements and *trans*-acting splicing factors (**Table 2**) 23, 39.

First, circRNAs can compete with linear splicing through RNA Pol II. A mutation in RNA Pol II that increases splicing efficiency produces remarkably lower numbers of circRNAs, which indicates strong competition between circRNA production and linear splicing 54. In proliferating cells, the splicing efficiency should be higher than that in non-proliferating cells, so as to meet the need for cell division and RNA synthesis. Thus, proliferating cells may have lower proportions of circRNAs because more linear RNAs are needed. However, it is unknown how circRNAs modulate RNA Pol II. For ElciRNAs, a study published in 2015 revealed that circEIF3J and circPAIP2 interact with RNA Pol II and U1 snRNP in the nucleus, subsequently promoting the transcription of their parental genes 42. Furthermore, ci-ankrd52 and ci-sirt-7 were found to interact with the RNA Pol II elongation complex, and suppression of these circRNAs led to decreased transcription levels of the ankyrin repeat proteins, Ankyrin Repeat Domain 52 (ANKRD52) and Sirtuin 7 (SIRT7), respectively 33. Thus, all of the evidence to date indicates that RNA Pol II can regulate the biosynthesis of circRNAs.

Second, a series of studies illustrated that RBPs can regulate circRNA circularization in different systems and organisms. These RBPs include double-stranded RNA (dsRNA)-specific Adenosine Deaminases Acting on RNA (ADAR), QKI, FUS, nuclear factors NF90/NF110, DExH-box helicase 9 (DHX9), Heterogeneous Nuclear Ribonucleoprotein L (HNRNPL), RNA-Binding Motif 20 (RBM20), Muscleblind protein (MBL)/vertebrate homolog Muscleblind-like protein 1 (MBNL1), Epithelial Splicing Regulatory Protein 1 (ESRP1), and serine/arginine (SR)-rich proteins 61-65. FUS binds to introns to flank the back-splicing junctions during back-splicing reactions, which affects circRNA expression in murine embryonic stem cell-derived motor neurons 61. Ashwal-Fluss et al. were the first to identify the involvement of the MBL protein in exon circularization based on the circularization rates of bracketed exons 54. The RBPs QKI and RBM20 have also been shown to increase circRNA expression and to even induce their formation from otherwise linear transcripts via binding to specific intronic motifs. Binding of QKI to motifs present in both of the intronic regions close to the circularized exons could facilitate RNA looping and back-splicing through protein-protein dimerization 40. Abdelmohsen et al. first reported competition between circPABPN1 and its cognate mRNA for the RPB, Hu-antigen R (HuR) 53. On the other hand, ADAR enzymes, which prevent activation of the innate immune system by editing adenosine to inosine in endogenous dsRNA, suppress the biogenesis of circRNAs that rely on base pairing between inverted repeats 66. Thus, NF90/NF110 and HNRNPL/SRs act in combinatorial and coordinated ways in circRNA biogenesis 67.

It is worth noting that the effects of RBPs on the regulation of circRNA formation vary widely depending on the types of circRNA, tissue or cell, as well as biological circumstances. One RBP may have two distinct effects on circRNAs because different binding elements flanking the circRNAs can interact with different functional domains of the RBP. The expression of RBPs is also spatiotemporally specific, which contributes to the diverse expression of circRNAs in different cells under different pathophysiological circumstances. Thus, RBPs may serve as activators or inhibitors of the formation of circRNAs and, therefore, these proteins regulate circRNA expression levels in different ways and through multiple mechanisms.

Third, similar to mRNAs, recent studies revealed that circRNAs are also regulated by transcription factors. For example, c-Myb and TWIST1 (a basic helix-loop-helix transcription factor) can bind to the promoters of circHIPK3 and Cul2 circRNA, respectively, and up-regulate their expression 21, 68; after activation of the *Cul2* promoter, the levels of *Cul2* pre-mRNA and circRNA increase while the levels of *Cul2* mRNA and protein unexpectedly decrease. However, the fate of mRNAs sharing the same promoter with circRNAs is unknown. Further research is necessary to explore the underlying mechanisms involved.

#### Degradation

CircRNAs are not subject to many of the canonical RNA degradation pathways. Currently, very little is known about the mechanisms of how circRNAs are degraded in vivo. In theory, the degradation of circRNAs can be initiated by an endonuclease followed by a combination of exo- and endonucleases. Currently, very little is known about the mechanisms and rates at which circRNAs are degraded *in vivo*. The first evidence of natural circRNA degradation via endonuclease activity was found *in vitro* using RNase H and Rrp44 69-71. However, circRNAs were also shown to be degraded by artificial shRNA/siRNA-based systems efficiently *in vitro* 72. Recently, the RNA modification, m^6^A, as well as poly(I:C) stimulation were shown to play a role in activating the endoribonuclease, RNase L, and in the degradation of circRNAs 15, 73. Interestingly, other models of degradation have recently been uncovered. For example, under normal conditions, circPOLR2A binds to double-stranded RNA (dsRNA)-activated protein kinase (PKR), while under the virus-infected condition, PKR is activated and released from the circRNA, allowing RNase L to recognize and degrade the corresponding circRNA (**Table 2**) 15. The miR-671-targeting site of CDR1as/ciRS-7 can trigger its cleavage in an Ago2 slicer-dependent manner 30. The GW182 protein, which consists of an Ago-binding domain (ABD), a ubiquitin-associated domain (UBA), a glutamine-rich domain (Q-rich), a middle region (Mid), an RNA-recognition motif (RRM), and a C-terminal region (C-term), is also involved in the degradation of many circRNAs in species from *Drosophila* to humans 74. Although not obligatory, these specific domains can accelerate circRNA degradation. In addition to degradation, circRNAs may also be eliminated from cells by exocytosis or through the activity of exosomes 75.

### Binding site-based *vs.* tertiary structure-based modes of circRNA-protein interaction

**Figure 2:**
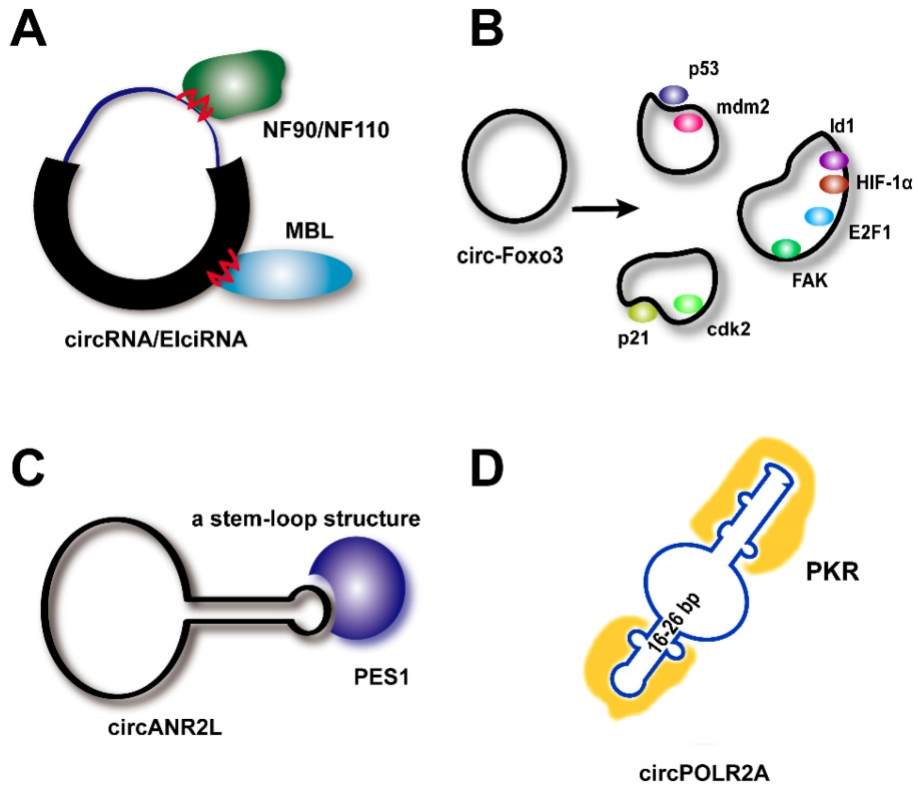
**Binding site-based *vs.* tertiary structure-based modes of circRNA-protein interactions. (A)** Circ-Mbl contains several binding sites for the mannose-binding lectin (MBL) protein. Nuclear factor (NF) complexes 90/110 (*NF90/NF110*) promote circRNA production in the nucleus by associating with intronic RNA pairs and interacting with mature circRNAs in the cytoplasm through binding sites. **(B)** Circ-Foxo3 displays a variety of tertiary structures in various cell/tissue environments (**see the text**). **(C)** CircANRIL appears to form a stem-loop structure that mimics rRNA and binds to Pescadillo homolog 1 (PES1). **(D)** CircPOLR2A tends to form 16-26 bp of imperfect base-paired RNA duplexes to inhibit double-stranded RNA (dsRNA)-activated protein kinase (PKR). cdk2: cell division protein kinase 2. E2F1: E2F transcription factor 1. FAK: focal adhesion kinase. HIF-1α: hypoxia-inducible factor 1α. Id1: inhibitor of differentiation 1. Mdm2: Mouse double minute 2 protein. p21: cyclin-dependent kinase inhibitor 1.

RNAs usually interact with proteins through electrostatic interactions, hydrogen bonding, hydrophobic interactions, and base stacking in a manner similar to that of DNA-protein interactions 82. Based on bioinformatic analyses, many circRNAs are predicted to harbor RNA-binding protein sites (**Figure 2A**) 83. Since 2013, circRNAs have broadly been reported to bind to proteins and act as protein sponges, similar to their known activity as miRNA sponges.

In 2014, Ashwal-Fluss et al. demonstrated that circ-Mbl contains several binding sites for the MBL protein, thereby regulating both circ-Mbl and MBL biogenesis 54. Since then, a series of studies have proven this concept, particularly for the RBPs, including the ADAR, QKI, FUS, NF90/NF110, DHX9, HNRNPL, RBM20, ESRP1, and SR proteins, which all have been proposed to contain binding motifs for their corresponding circRNAs. An RBP can also have multiple target RNAs. Schneider et al. found 34 circRNAs to be associated with IMP3 (IGF2BP3, Insulin-like Growth Factor 2-Binding Protein 3) 84. RBPs contain various structural motifs, such as the RNA-recognition motif (RRM), dsRNA-binding domain, zinc finger domain, and others 85, and several of these may be involved in circRNA binding.

Recently, it was shown that circ-Amotl1 binds to PDK1 and AKT1, and putative PDK1- and AKT1-binding sites were then identified 46. Binding of these molecules leads to AKT1 phosphorylation and nuclear translocation, while oligonucleotides complementary to circ-Amotl1 can reverse the effects of exogenous circAmotl1 46.

The existing methods used to explore the mechanisms of protein-circRNA binding fall into two categories: prediction of the circRNA-binding sites on the protein or prediction of the protein-binding sites along the circRNA. The prediction of protein-binding sites along the circRNA chains is the more difficult approach because of limited information regarding circRNA sequences. The prediction of protein-RNA binding sites is essentially a classification problem, involving both unique representations for sequences and classification models. The cornerstone of the theory is *K*-tuple nucleotide composition, which is primarily based on the proportion of A (adenine), G (guanine), C (cytosine), and U (uracil) bases 86, 87. There are many useful databases based on this theory to predict and explore the role of RNA-protein interactions, such as CircInteractome (Circular RNA Interactome, https://circinteractome.nia.nih.gov/index.html).

Zhang and colleagues found that the binding sites of circRNAs for some RBPs exhibit common patterns, such as the “GAAGAAG'” motif common among several RBPs including Argonaute 2(Ago2), α-ketoglutarate-dependent dioxygenase alkB homologue 5 (ALKBH5), cell cycle-associated protein 1 (CAPRIN1), lin-28 homolog B (LIN28B), and insulin‑like growth factor 2 mRNA‑binding protein 3 (IGF2BP3) 88. Despite these common motifs, for the same RBP, binding sites on circRNAs and linear RNAs can have large sequence diversity. Interestingly, when FUS binds to circRNAs, the specificity of RBP binding becomes much higher than that for linear RNAs 89.

However, the circRNA-protein interaction theory based on binding sites does not explain all of the interactions observed to date. Reports show that circRNAs without any predicted binding sites also have the capacity to interact with proteins 90. Increasingly, studies are revealing that certain proteins are able to bind to different circRNAs 91, 92, whereas several circRNAs can also dynamically bind to different proteins 22, 44. It appears that the tertiary structure of circRNAs among other factors may contribute to these interactions. Indeed, protein interactions with other types of RNA are known to be significantly influenced by the tertiary structure of the RNA molecules 19. The distinct tertiary structure of circRNAs may entail a more complex protein-binding process than previously thought, and further studies are needed to reveal the precise mechanisms thereof.

Circ-Foxo3 has been shown to bind to a variety of proteins in different cells and under different conditions (**Figure 2B**). For example, circ-Foxo3 interacts with anti-stress protein ID-1, anti-stress protein FAK, HIF-1a, and the transcription factor, E2F1, thereby retaining these proteins in the cytoplasm and leading to cellular senescence 22. This circRNA also enhances the interaction between p21 and Cdk2 in the cytoplasm, thereby preventing Cdk2 interaction with cyclins A and E and subsequently blocking cell cycle progression 44. Du and colleagues concluded that because factors in the cellular environment, such as solvents and metal ions, have a strong influence on the dynamic tertiary structure of circRNAs, circRNA tertiary structure may be different in various cell lines, tissues, and developmental stages 19. Thus, these circRNAs could display a variety of different functions in different tissues or development stages by binding to different functional proteins. However, the extent of this structural fluidity has not yet been revealed or proven directly, and further, in-depth studies are still needed. Current evidence supporting this theory is that the circRNA, circDNMT1, shows significant functional diversity by binding to a variety of tissue-specific proteins 49, 56.

Recently, further mechanistic details were uncovered (**Figure 2C-D**). CircANRIL appears to form a stem-loop structure that mimics that of rRNA and binds to PES1, thus blocking its interaction with the PeBoW complex (Pes1, Bop1, and WDR12), which is a key regulator of the biogenesis of the 60S ribosomal subunit 47. Furthermore, a study in 2019 showed that an endogenous circRNA (circPOLR2A) tends to form 16-26 bp of imperfectly base-paired RNA duplexes that inhibit the function of double-stranded RNA (dsRNA)-activated protein kinase (PKR) in innate immunity 15. That study further confirmed that overexpression of circSMARCA5, which lacks an intra-dsRNA region, does not suppress PKR activation upon poly(I:C) treatment (circRNAs suppress PKR activity depending on their dsRNA-mediated activation). These results showed that the tertiary structure of circRNAs indeed plays an extremely important role. However, further questions are raised. When observing circRNAs *in vitro*, their tertiary structures may change, but there is no method available with which to reveal their real dynamic interactions *in situ*. Furthermore, since circRNAs are stable and conserved, deformation of the tertiary structure should be more difficult. How, therefore, do circRNAs bind to different proteins as decoys and scaffolds? The research field of circRNAs is still in its infancy, and there is much more work to be carried out and exciting discoveries to be made.

### The functions of circRNA-protein interactions

#### Protein sponges

Even though most circRNAs do not contain multiple protein-binding sites, many circRNAs are able to act effectively as protein sponges. For example, QKI binds to sites flanking circRNA-forming exons, thereby regulating circRNA formation during Epithelial-mesenchymal transition (EMT), and the insertion of QKI-binding sites into linear RNA can induce exon circularization 40. Additionally, circ-Amotl1 binds to PDK1 and AKT1 through binding sites to mediate wound repair 93 (**Figure 1D**).

#### Protein decoys

In their function as protein decoys, circRNAs cooperate with the target protein at the appropriate cellular site to change the protein's routine physiological function. For example, circ-Amotl1 is able to bind and retain c-Myc in the nucleus, where it stabilizes c-Myc, up-regulates its target genes, and leads to increased cell proliferation, reduced apoptosis, and a highly tumorigenic phenotype 94. Furthermore, intronic lariats can serve as a sort of bait/pool for TDP43 (TAR-DNA binding protein 43) in the cytoplasm 95. CircPABPN1 is suggested to sequester HuR, thereby serving as a decoy for HuR and impairing PABPN1 translation 53. CircPOLR2A and circDHX34 serve as a molecular reservoir for NF90 and/or NF110 in normal cells prior to viral infection 78. Thus, different circRNAs are able to bind to different proteins in various tissues and under various circumstances. The only common feature is that RNAs have the capacity to interact with proteins specifically and with high affinity, suggesting that most circRNAs may play an antagonistic role with regards to the normal physiological effects of their target proteins and that these RNAs may comprise an alternative form of self-regulation under stressful conditions (**Figure 1E**).

#### Protein scaffolds

CircRNAs can also function as scaffolds facilitating contact between two or more proteins. Some circRNAs, such as circ-Amotl1 and circ-Foxo3, function as protein scaffolds to facilitate the co-localization of enzymes and their substrates. Circ-Amotl1 was found to act as a scaffold during interactions with PDK1 and AKT1 to facilitate their nuclear translocation, which is important for cell proliferation and survival 46. In a study by Du et al., circ-Foxo3 was observed to function as a scaffold for several proteins. For example, circ-Foxo3 can bind to p53 and the E3 ubiquitin-protein ligase, Mdm2, to promote Mdm2-induced ubiquitination and the subsequent degradation of p53 45 (**Figure 1F**).

#### Protein recruitment

CircRNAs may also recruit specific proteins to certain cellular locations, as exemplified by the circRNA, FECR1, which recruits Ten-Eleven Translocation methylcytosine dioxygenase 1 (TET1) to the promoter region of FLI1, its host gene, leading to the demethylation of CpG sites and active transcription 56. Furthermore, circ-Amotl1 recruits the Signal Transducer and Activator of Transcription 3 (STAT3) from the cytoplasm to the nucleus and stabilizes its binding to the *Dnmt3a* promoter 93, while circMYBL2 regulates FMS-Like Tyrosine kinase-3 (FLT3) translation by recruiting Polypyrimidine Tract-Binding Protein 1 (PTBP1) to promote internal tandem duplication of the *FLT3* gene during acute myeloid leukemia progression 96 (**Figure 1G**).

#### Translation

CircRNAs were originally believed to be untranslatable. However, circRNAs are mostly cytosolic and originate from protein-coding exons, giving rise to the question of whether they could be loaded onto ribosomes and translated into proteins. It has since been shown that circRNAs can be translated both *in vitro* and *in vivo* 97, 98, although endogenous translation of circRNAs has been only indirectly tested thus far 99 (**Figure 1H**). There are several well-studied protein-coding circRNAs: circ-ZNF609 100, circ-FBXW7 38, circSHPRH 101, cirPINTexon2 102, circMbl 103, and the recently reported circ-AKT3 104 (**Table 3**). These studies suggest that an internal ribosome entry site (IRES) and an open reading frame (ORF) are necessary components for circRNA protein translation 105, 106. Furthermore, N^6^-methyladenosine, the most abundant base modification of RNA, can promote efficient initiation of protein translation from circRNAs in human cells 107.

Some specific circRNAs are also associated with translating ribosomes relating to cap-independent translation 35. Untranslated regions of ribo-circRNAs (cUTRs) allow cap-independent translation *in vitro* and *in vivo,* which is independent of IRES sequences. Moreover, the existence of a specific sequence in 4E-BP and FOXO can enhance their translation via the circMbl cUTR. CircMbl1 and the putative circMbl1-encoded peptide are present in synaptosome fractions, as determined by mass spectrometry 103.

The micro-proteins encoded by circRNAs are relatively short at around 146-344 amino acids in length 108. All circRNA-encoded proteins have been found in metabolically active cells such as cancer cells or myoblasts (**Table 3**), which suggests that the translation of circRNAs is a rescue measure or a supplement to meet the extra requirements of these cells; the translated proteins may also have certain functions in such cells. Although the physiological functions of most of these proteins have not yet been identified, it is likely that they share some of the functional abilities of their full-length protein counterparts encoded by the linear forms of the transcripts 108. For example, the circ-FBXW7 encodes micro-protein FBXW7-185aa, which competes with FBXW7 to bind USP28 (an inhibitor of c-Myc degradation) to promote c-Myc degradation 38. However, there are still many unresolved questions and much debate regarding this aspect of circRNAs. For example, which circRNAs are translated and the circumstances under which these circRNAs are translated remain unclear.

### Approaches used to detect circRNA-protein interactions

Currently, the interactions between circRNAs and proteins are mainly analyzed by RNA pull-down assays or through RNA immunoprecipitation (RIP). Genome-wide profiling, locus-specific profiling, and visualization of circRNAs are also used to discover, profile, and understand the biogenesis and functions of circRNAs.

#### RNase protection assay (RPA)

**Figure 3:**
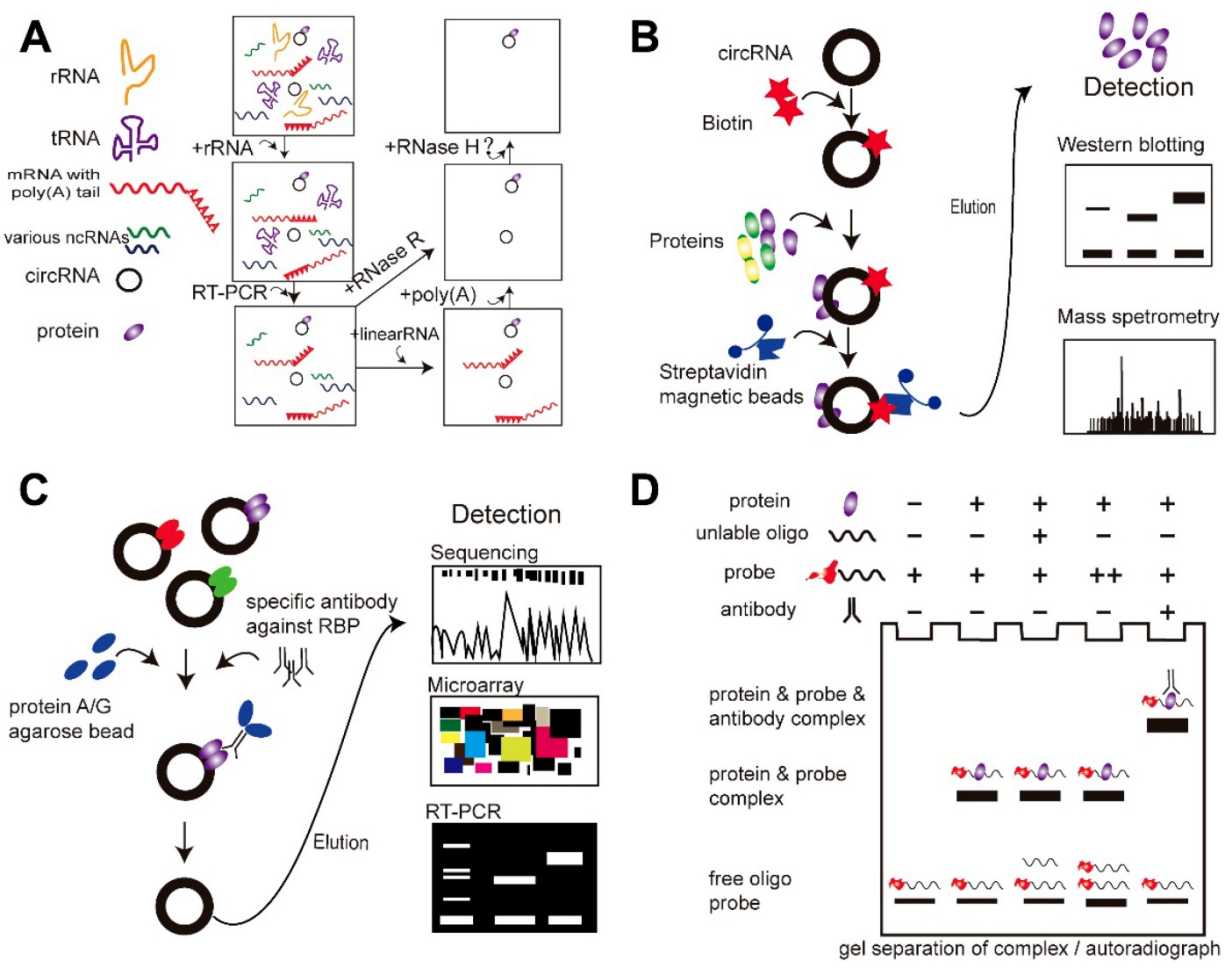
** The main approaches currently used to detect circRNA-protein interactions. (A)** RNase protection assay (RPA). **(B)** RNA pull-down assay. **(C)** RNA immunoprecipitation (RIP) assay. **(D)** Electrophoretic mobility shift assay (EMSA).

RPA is a powerful method for detecting RNA and RNA fragments in cell extracts. Different RNA enzymes have corresponding RNA substrates; thus, the target RNA can be purified continuously allowing the target circRNAs to be obtained. CircRNAs can be enriched by excluding rRNA, tRNA, poly(A)^+^ RNAs, and linear RNAs with the use of their corresponding RNA enzymes or with PCR. The circular nature of candidate circRNAs can be validated by treating total RNA with RNase R, which degrades linear RNAs but not circRNAs. RPA assays can also be used to map protein-RNA interactions: when a protein binds to the RNA at the target sequence, it will prevent cleavage by RNase H and indicate a site of interaction between the protein and RNA 71 (**Figure 3A**).

#### RNA pull-down assay

This assay uses probes for known circRNAs to investigate putative protein-binding partners. These DNA oligo probes are conjugated with streptavidin-coated magnetic beads. In brief 44, cell cultures are washed in ice-cold phosphate-buffered saline and lysed in co-IP buffer, followed by incubation with biotinylated DNA oligo probes at room temperature. Streptavidin-bound magnetic beads are then added to each binding reaction. In this system, the probes bind to the circRNAs, and binding of the streptavidin-coated magnetic beads allows the target circRNAs and any bound protein partners to be pulled down together. The beads are then washed, and the bound proteins are confirmed by western blotting or mass spectrometry. During this process, there are several important steps: (1) the circRNAs must be purified with RNAse R; (2) the probes should specifically target the back-splicing junction region of the circRNAs; and (3) because of their complex tertiary structures, circRNAs may pull down non-specific binding proteins, and tighter environmental controls mimicking physiological conditions should be considered (**Figure 3B**).

#### RNA immunoprecipitation (RIP)

RNA-binding protein immunoprecipitation assay (RIP) followed by circRNA sequencing is another feasible strategy for analyzing circRNA-protein interactions 44, 84. In contrast to the RNA pull-down assay, RIP detects the RNAs by targeting the protein. Briefly, in functional assays, cells are washed in ice-cold PBS, lysed in co-IP buffer, and incubated with a primary antibody targeting the protein of interest. A total of 50% slurry of Protein A Sepharose is added to each sample, and the mixtures are incubated. The pellets are washed with PBS and resuspended in TRI Reagent. The eluted co-precipitated RNA in the aqueous solution is then subjected to qRT-PCR analysis to demonstrate the presence of the circRNA binding partners (**Figure 3C**).

#### Electrophoretic mobility shift assay (EMSA)

EMSA is a common affinity electrophoresis technique used to study protein-DNA and protein-RNA interactions 110. This procedure can determine whether a protein or mixture of proteins is capable of binding to a given RNA sequence, and it can sometimes indicate whether more than one protein molecule is involved in the binding complex. The circRNAs and protein are incubated and the resulting mixtures are subjected to electrophoresis under native conditions through a polyacrylamide or agarose gel. After electrophoresis, the distribution of species containing circRNAs is determined by western blotting with a specific antibody against the protein of interest. In general, circRNA-protein complexes migrate more slowly than the corresponding free circRNA (**Figure 3D**).

#### Fluorescence *in situ* hybridization (FISH/ISH)

FISH/ISH can be used to detect circRNA-protein binding with the use of DNA oligo probes and specific, fluorescently labeled antibodies to determine the position of binding 19. In brief, cells or frozen tissue sections are fixed and then permeabilized to allow target accessibility. A fluorescently labeled target-specific probe composed of 20 base pairs hybridizes to the target RNAs. Signal amplification is achieved via a series of sequential hybridization steps. At the end of the assay, the tissue samples are visualized under a fluorescence microscope.

## Conclusions and future perspectives

Owing to RNA-Seq and other advanced technologies (e.g., single-cell RNA-Seq 111, 112), circRNAs have become a major topic of research because of their variety of biological functions including gene expression regulation, mRNA competition, protein interaction, and protein coding. In this review, we have summarized the biogenesis, characteristics, protein interactions, and translation of circRNAs, to provide an overview of current knowledge regarding the functional mechanisms of circRNA-protein interactions.

Under conditions of limited space, circular molecules may be able to bind to more proteins, enabling them to act as protein sponges, decoys, stabilizers, scaffolds, protein recruiters, and as protein products themselves. In addition, circular scaffolds such as circRNAs may be crucial for proteins to assemble in a specific spatial orientation. The evidence indicates that circRNAs can bind to proteins not only through binding motifs, but also through interactions based on their tertiary structure. Interestingly, circRNA-protein interactions affect both partners in a dynamic manner 19. Even though thousands of putative circRNAs in different tissues and diseases have been identified and many functional proteomic studies of circRNAs have been performed, research aimed at directly revealing the mechanism of circRNA-protein interactions is limited, which may be due to current technological limitations. A lack of relevant and effective methods for *in vivo* research may be an important obstacle in this field.

Here, we also reviewed the methods used to study the interactions between circRNAs and proteins, and the approaches employed can be classified as either circRNA-based or protein-based. However, limited research has been carried out to determine how the tertiary structures of the circRNA and protein partners contribute to their interactions *in vivo*.

The circRNA research field has brought many surprising findings, such as the fact that circRNAs have the potential to encode proteins or polypeptides 38, 100-103, that circRNAs can be extracted from the cell or organism via exosomes 113, and/or can act as biomarkers 114, 115.

Arguably, the most attractive area in circRNAs research is related to exosomes. Exosomes have been shown to reduce the accumulation of circRNAs and to assist with circRNA clearance, providing evidence of circRNA degradation 116. Furthermore, exosomes are not only messengers between cells because of their ready access to bodily fluids, but because they can also carry circRNAs, thereby facilitating the transfer of biological information and material to target cells 113. Furthermore, exosomes can protect circRNAs from clearance in cells. The mechanisms by which circRNAs are enriched during exosome formation are, however, unknown.

Another interesting area is immunology 117. As recently as 2017, Chang et al. reported that cells can recognize both endogenous circRNAs produced in cells and exogenous circRNAs synthesized *in vitro* with the Retinoic acid-Inducible Gene 1 protein (RIG-1) (**Figure 1K**). However, RIG-1 activates autoimmune pathways in response to viral infections only after recognizing exogenous circRNAs 118. In contrast, in March 2019, Anderson et al. reported that exogenous synthetic circRNAs transfected into cells did not stimulate immune pathways 119. They proposed that the previously reported immune reaction to circRNAs was due to insufficient purity of the samples, which may have arisen from the non-specific effects of the presence of linear RNAs with a 5' phosphate group. However, this was later refuted by Chang and colleagues who validated their previous results 120. They showed that the m^6^A RNA modification of human circRNAs abrogates immune gene activation and the adjuvant activity of innate immunity. Unmodified circRNA, but not m^6^A-modified circRNA, directly activates the RNA pattern recognition receptor, RIG-1. In addition to exogenous circRNA, endogenous circRNA can also cause an immune response. Endogenous circRNAs can be used as “molecular indicators” of antiviral proteins such as NF90/NF110, which prompt an antiviral immune response by decoying virus proteins in the nucleus for export into the cytoplasm to inhibit virus replication 67.

Although there are many existing conflicts and controversies in circRNA research 16, 28, we hope to see increasing research on circRNAs, as these RNAs are proposed to comprise a new generation of predictive biomarkers and therapeutic targets with clinical promise.

## Figures and Tables

**Table 1:** Known functions of circRNA-protein interactions.

circRNAs	Interacting proteins	Biological functions	Types of cell/tissue	Cytoplasm/nucleus	Ref.
**circFOXO3**	P21,CDK2	inhibits cell cycle progression	mouse fibroblast NIH3T3	cytoplasm	44
**circFOXO3**	ID-1,E2F1,FAK,HIF1α	cardiac senescence	cardiomyocytes/cardiac fibroblasts	cytoplasm	22
**circFOXO3**	MDM2,P53	induces apoptosis	breast cancer cell	cytoplasm	45
**circAmotl1**	PDK1,AKT1	promotes cell survival and protects against doxorubicin-induced cardiomyopathy	Cardiomyocytes	cytoplasm	46
**circANRIL**	PES1	impairs pre-rRNA to maturate and ribosome biogenesis in atherosclerosis	vascular tissue/smooth muscle cell	cytoplasm	47
**circHECTD1**	HECTD1/ZC3H12A	mediates macrophage activation and fibroblast activation and migration	Macrophage	no mentions	48
**circDNMT1**	P53,AUF1	promotes cell proliferation by activating autophagy	breast cancer cell	cytoplasm	49
**circSMARCAS5**	SRSF1	acts as tumor suppressor: anti-angiogenic and cells migration	Glioblastoma	no mentions	50, 51
**circMTO1**	TRAF4	inhibits cell proliferation	breast cancer cell	no mentions	52
**circPABPN1**	HuR	suppresses PABPN1 translation	HeLa cell	cytoplasm	53
**circMbl**	MBL	regulates mbl functions, translation and neuronal functions	nerve cell	cytoplasm	54
**cia-cGAS**	cGAS	protects long-term haematopoieyic stem cells from exhaustion	haematopoieyic stem cell	nucleus	55
**FECR1**	TET1	recruits TET1 to the promoter region of its own host gene and up-regulation of FLI1	breast cancer	nucleus	56
**ci-ankrd52**	RNA Pol II	enhances the RNA Pol II transcription to up-regulate the its parental gene	PA1 cell	nucleus	33
**circPAIP2**	RNA Pol II	enhances the RNA Pol II transcription to up-regulate the its parental gene	central nervous system	nucleus	42
**circARSP91**	AR-ADAR1	suppresses tumor growth	hepatocellular carcinoma	cytoplasm	57
**circARSP91**	UL16 binding protein 1 (ULBP1)	enhances the cytotoxicity of natural killer cells against hepatocellular carcinoma	hepatocellular carcinoma	cytoplasm	58
**circZKSCAN1**	FMRP	against CCAR1 complex to induced tumor quiescence *in vivo*	hepatocellular carcinoma cancer stem cells	cytoplasm	59

**Table 2:** Studies on RBPs and other proteins regulating circRNA biogenesis and degradation

RBPs and other proteins	Target circRNAs	Effects	Experimental/disease model	Ref.
**RBM20**	circRNAs that originate from the I-band of the Titin gene	Biogenesis	Dilated cardiomyopathy	62
**RBM3**	SCD (stearoyl-CoA desaturase)-circRNA 2	Biogenesis	Hepatocellular carcinoma	76
**ADAR**	circARSP91	Biogenesis	Hepatocellular carcinoma	57
**QKI**	specific circular RNAs derived from Titin (formin homology 2 domain containing 3) and Striatin (calmodulin-binding protein 3)	Biogenesis	Doxorubicin-Mediated Cardiotoxicity	77
**QKI**	circRNAs:POLE2, SMARCA5, OXNAD1, SHPRH, SMAD2, ATXN2, DOCK1, GNB1	Biogenesis	Epithelial-mesenchymal transition	40
**NF90/NF110**	circPOLR2A, circDHX34, circPDE3B	Biogenesis	Viral infection model in HeLa cells	78
**DHX9**	circular RNAs containing inverted-repeat Alu elements	Biogenesis	Human/mouse embryonic stem cells	65
**HNRNPL**	139 significantly up-regulated and 93 down-regulated circRNAs upon HNRNPL knockdown	Biogenesis/Degradation	Prostate Cancer	63
**MBL/MBNL1**	circMbl	Biogenesis	Drosophila S2 cells and murine homolog	54
**FUS**	136 circRNAs varied significantly with 111 were downregulated upon FUS knockdown	Biogenesis/Degradation	Amyotrophic lateral sclerosis	54
**ESRP1**	circBIRC6	Biogenesis	human embryonic stem cells	79
**c-Myb**	circHIPK3	Biogenesis	Colorectal cancer	80
**TWIST1**	Cul2 circRNA	Biogenesis	Epithelial-mesenchymal transition	81
**hnRNP and SR proteins**	Laccase2 circular RNA	Biogenesis	Drosophila S2 and DL1 cell	39
**RNA Pol II and U1 snRNP**	circEIF3J, circPAIP2	Biogenesis	HEK293 cells	33
**RNA Pol II and U1 snRNP**	ci-ankrd52, ci-sirt-7	Biogenesis	human cells (GM12878, HUVEC, HepG2, NHEK, HeLa S3, and K562 cell lines)	42
**RNase L**	Global circRNAs	Degradation	Systemic lupus erythematosus	15
**Rrp44**	Global circRNAs	Degradation	Yeast	70
**GW182**	circRNAs: CaMKI, Dbp80, ues, laccase2, Hapin, ps, pan, dati, PlexA, Ect4, mnb	Degradation	Drosophila DL1 or S2 cells	74
**PKR**	circPOLR2A	Degradation	Systemic lupus erythematosus	15
**Ago2**	CDR1as/ciRS-7	Degradation	HEK293 cell lines	30

**Table 3:** CircRNAs known to be translated into proteins.

circRNAs	Biological functions	Types of cell/tissue	Cytoplasm/nucleus	Ref.
**circ-ZNF609**	regulates myoblast proliferation	Myoblast	Cytoplasm	100
**circ-FBXW7**	tumour inhibitor	cancer cell	Cytoplasm	38
**circPINTexon2**	tumour inhibitor	cancer cell	Cytoplasm	102
**circSHPRH**	tumour inhibitor	cancer cell	Cytoplasm	101
**circMbl3**	translation of circRNAs	cancer cell	Cytoplasm	103
**circ-AKT3**	tumour inhibitor	cancer cell	Cytoplasm	104
**circCFLAR**	unknown	myoblast	Cytoplasm	109
**circSLC8A1**	unknown	myoblast	Cytoplasm	109
**circMYBPC3**	unknown	myoblast	Cytoplasm	109
**circRYR2**	unknown	myoblast	Cytoplasm	109
